# Effects of Si-Miao-Yong-An decoction on myocardial I/R rats by regulating gut microbiota to inhibit LPS-induced TLR4/NF-κB signaling pathway

**DOI:** 10.1186/s12906-023-04013-9

**Published:** 2023-06-02

**Authors:** Yuting Cui, Fangyuan Zhang, Weiming Xu, Ziyun Li, Jiaxi Zou, Ping Gao, Jingqing Hu

**Affiliations:** 1 Changchun University of Chinese Medicine, Changchun, China; 2 Institute of Basic Theory for Chinese Medicine, China Academy of Chinese Medical Sciences, Beijing, China; 3 China Science and Technology Development Center for Chinese Medicine, Beijing, China; 4 The First Affiliated Hospital of Henan University of CM, Zhengzhou, China; 5 School of Acupuncture and Tuina, School of Regimen and Rehabilitation, Nanjing University of Chinese Medicine, Nanjing, China; 6 School of Basic Medical Sciences, Chengdu University of Traditional Chinese Medicine, Chengdu, China; 7 Affiliated Hospital of Integrated Traditional Chinese and Western Medicine, Nanjing University of Chinese Medicine, Nanjing, China

**Keywords:** Ischemia/Reperfusion, Si-Miao-Yong-an, Gut microbiota, Intestinal permeability, TLR4/NF-κB

## Abstract

**Background:**

Coronary Artery Disease (CAD) is primarily caused by inflammation which is closely linked to the gut microbiota. Si-Miao-Yong-An (SMYA) decoction is a traditional Chinese herbal formula with anti-inflammatory properties that found to be effective against CAD. However, it is still unclear whether SMYA can modulate gut microbiota and whether it contributes to the improvement of CAD by reducing inflammation and regulating the gut microbiota.

**Methods:**

The identification of components in the SMYA extract was conducted using the HPLC method. A total of four groups of SD rats were orally administered with SMYA for 28 days. The levels of inflammatory biomarkers and myocardial damage biomarkers were measured through ELISA, while echocardiography was used to assess heart function. Histological alterations in the myocardial and colonic tissues were examined following H&E staining. Western blotting was performed to evaluate protein expression, whereas alterations in gut microbiota were determined by 16 s rDNA sequencing.

**Results:**

SMYA was found to enhance cardiac function and decrease the expression of serum CK-MB and LDH. SMYA was also observed to inhibit the TLR4/NF-κB signaling pathway by downregulating the protein expression of myocardial TLR4, MyD88, and p-P65, leading to a reduction in serum pro-inflammatory factors. SMYA modified the composition of gut microbiota by decreasing the Firmicutes/Bacteroidetes ratio, modulating Prevotellaceae_Ga6A1 and Prevotellaceae_NK3B3 linked to the LPS/TLR4/NF-κB pathway, and increasing beneficial microbiota such as Bacteroidetes, Alloprevotella, and other bacterial species. Moreover, SMYA was found to safeguard the intestinal mucosal and villi structures, elevate the expression of tight junction protein (ZO-1, occludin), and reduce intestinal permeability and inflammation.

**Conclusions:**

The results indicate that SMYA has the potential to modulate the gut microbiota and protect the intestinal barrier, thereby reducing the translocation of LPS into circulation. SMYA was also found to inhibit the LPS-induced TLR4/NF-κB signaling pathway, leading to a decrease in the release of inflammatory factors, which ultimately mitigated myocardial injury. Hence, SMYA holds promise as a therapeutic agent for the management of CAD.

**Supplementary Information:**

The online version contains supplementary material available at 10.1186/s12906-023-04013-9.

## Introduction

Angiocardiopathies are the primary cause of death globally, with ischemic cardiopathies accounting for 49.2% of all cardiovascular deaths [1]. The incidence and mortality of Coronary Artery Disease (CAD) have increased worldwide [2]. CAD is the most common ischemic cardiomyopathy caused by obstruction, spasm, or narrowing of the cardiac lumen due to coronary atherosclerosis. This condition impairs the myocardial supply of oxygen and blood, leading to myocardial ischemia or necrosis [3]. Myocardial ischemia/reperfusion (I/R) injury causes the death of myocardial cells, which triggers the recruitment of inflammatory cells to clear cell debris. Conversely, undue inflammation exacerbates myocardial damage [4, 5]. Inflammatory factors are one of the major causative factors of myocardial I/R injury [6]. Inflammation plays a critical role in CAD development [7]. Inflammatory markers such as C-reactive protein (CRP), homocysteine (Hcy), and Interleukin-6 (IL-6) are significantly elevated in CAD patients. However, no drugs are available to act on these inflammatory factors and inhibit them, ultimately treating CAD. Therefore, novel treatment strategies focusing on the inflammatory aspect of CAD need to be explored.

The gut microbiota is known as the “second human genome.” Additionally, billions of bacterial species reside in the intestinal tract, making up the largest microfloral ecosystem in the human body [8, 9]. Recent studies suggest that the gut microbiota and its metabolites are involved in the development of angiocardiopathy [10]. Gut microfloral metabolites, such as Lipopolysaccharide (LPS), Trimethylamine oxide (TMAO), and short-chain fatty acids (SCFA), affect CAD development through the immune response, inflammatory stimulation of nuclear factor kappa-B (NF-κB), lipid metabolism, and bile acid metabolism [11–13]. LPS increases intestinal permeability, enters the systemic circulation, induces inflammation, and subsequently damages the cardiac muscle [14, 15]. Moreover, it activates TLR, thereby stimulating NF-κB signaling and secreting downstream inflammatory cytokines such as IL-6, IL-1β, IL-17A, and TNF-α [16], participating in the myocardial inflammatory response [17]. Therefore, therapeutic strategies that aim to improve the inflammatory state by utilizing the gut microbiota might act as a novel approach to treating CAD.

Herbs possessing antipyretic and detoxifying properties exhibit anti-inflammatory [18], hypoglycemic [16], hypolipidemic, and antithrombotic effects [19]. Si-Miao-Yong-An (SMYA) decoction comprises of four herbs: Jinyinhua (Flos Lonicerae; *Lonicera japonica* Thunb.), Xuanshen (Radix Scrophulariae; *Scrophularianingpoensis* Hemsl.), Danggui (Radix Angelicae; *Angelica sinensis* (Oliv.) Diels), and Gancao (Radix Glycyrrhizae; *Glycyrrhiza uralensis* Fisch.) [20]. SMYA has been shown to combat lipid deposition and inflammation and promote atherosclerotic plaque stability in a rabbit model [21]. SMYA improved cardiac I/R injury in rats, as evident from the improved ventricular volume and ejection fraction [22]. SMYA improved cardiac function, suppressed myocardial hypertrophy, apoptosis and inflammation, and exerted a preserving effect on cardiovascular disease [23]. An SMYA extract containing chlorogenic acid (CA), angoroside C (AGDC), cryptochlorogenic acid (CCA), neochlorogenic acid (NCA), isochlorogenic acid A (ICAA), isochlorogenic acid C (ICAC), harpagide (HPD), and sweroside (SRD) was found to effectively inhibit cardiac hypertrophy as well as the growth of H9c2 cells [24]. The primary active ingredients of SMYA are 3,5-dicaffeoylquinic acid (3,5-DiCQA) and angoriside C (AC), which are involved in improving cardiac function and reducing myocardial inflammation and fibrosis in rats [25]. Studies suggest that herbs with antipyretic and detoxifying properties could regulate gut microbiota and promote balance [26]. However, the mechanism by which SMYA regulates gut microbiota and inhibits inflammation to improve CAD remains unclear.

Hence, we aimed to investigate the role of SMYA in regulating gut microbiota and inflammation signaling pathways to elucidate the effective mechanism of SMYA in ameliorating CAD.

## Materials and methods

### Materials

For hematoxylin–eosin (H&E) staining, the G1120 kit from Solarbio Technology (Beijing, China) was used. The CK-MB (RS2011) and LDH (RS4044) reagents were obtained from InTec PRODUCTS, INC (Xiamen, China). ELISA kits for serum and colon tissue homogenate assays were procured from the North Institute of Biotechnology (Beijing, China) for TNF-α (R2784/1), IL-6 (R3021/1), IL-1β (R3001/1), IL-17A (R4421/1), and TLR4 (R3566/1). LPS (EC80545S) assay CETAL kits were obtained from Xiamen Bioendo Technology Co., Ltd (Xiamen, China). Primary antibodies for TLR4 (66350–1-Ig), MyD88 (23230–1-AP), P65 (10745–1-AP), Occludin (27260–1-AP), β-actin (66009–1-1 g), and Lamin B1(12987–1-AP) were purchased from Proteintech (UT, United States). Antibodies for p-P65 (3033S) and ZO-1 (13663) were acquired from Cell Signaling Technology (MA, USA). HRP-conjugated goat anti-mouse IgG (H + L) (A0216) and cell lysis buffer for Western blot and IP (P0013) were used as secondary antibodies and supplied by Beyotime Biotechnology (Shanghai, China). Protein marker (180–6003) and ECL substrate solution (high sensitivity) (180–501) were procured from Tanon (Shanghai, China). Aspirin (acetylsalicylic acid) (S17061) was acquired from Shanghai Yuanye Bio-Technology Co., Ltd (Shanghai, China).

### Preparation of SMYA

The herbs used in the study were procured from the Hefei HuarunSanjiu Pharmaceutical Co., Ltd. (Hefei, China). *L. japonicaeflos* was sourced from Shandong, China (production batch No. 20200903); *S. ningpoensisHemsl.* was sourced from Hubei, China (production batch No. 200313); *A. sinensis Radix* was sourced from Gansu, China (production batch No. 201028); and *Glycyrrhizae Radix et Rhizoma* was sourced from Neimenggu, China (production batch No. 20160068). About 30 g *L. japonicaeflos*, 30 g *S. ningpoensisHemsl*., 18 g A. sinensis Radix, and 18 g *Glycyrrhizae Radix* were diluted in distilled water at a 1:5 volume ratio of the total grams of the drug. The mixture was decocted for 50 min, and then distilled water was added in thrice the volume and decocted for 50 min again. After blending the two decoctions, their concentration was adjusted to a final value of 4 g/mL, equivalent to 4 g of SMYA decoction per mL of liquid. The drug was concentrated, filtered, and stored at –20 °C. Based on Clinical Medicine and the Laboratory of Pharmacology, a gavage dose of 8.64 g/kg/d was administered to the rats.

### High-performance liquid chromatography

The SMYA decoction that was prepared was converted into a dry powder using spin-drying. A quantity of 4 g of the SMYA powder was weighed and dissolved in 12 mL of distilled water. This solution was mixed with 28 mL of anhydrous ethanol in a volume of 40 mL and sonicated until it was fully dissolved. Standard compounds, including chlorogenic acid, neochlorogenic acid, isochlorogenic acid A, cryptochlorogenic acid, harpagoside, harpagide, imperatorin, liquiritin, isoliquiritin, glycyrrhizic acid, and others, were dissolved in methyl alcohol. The supernatant obtained after centrifugation (10000 rpm, 45 min) was used for analysis. The HPLC analysis was carried out using Waters Acquity I-Class UPLC-Waters SYNAPT G2-Si Q-TOF with Acquity UPLC HSS T3 (100 × 2.1 mm, 1.8 µm). The mass spectrum parameters were as follows: acquisition mode of Negative MSE, collection scope of 100–1500 Da, capillary voltage of 3.0 kV, ion source temperature of 120 °C, MS/MS energy range of 25–45. Chromatographic separation was achieved using a gradient elution solvent of methanol and formic acid, with a gradient elution of 0–20 min, B% 2%-35%; 20–30 min, B% 35%-95%. The flow velocity was set to 0.4 mL/min, and the column temperature was set at 45 °C. An injection volume of 2 µL was used for the determination of compound content in SMYA based on the retention times of standards.

### Animal model

The Animal Management Committee of the Institute of Basic Theory of Chinese Medicine (Beijing, China) approved the study (Permit Number: IBTTCMCAMS21–2104–04). Male Sprague Dawley rats weighing approximately 180 ± 20 g were purchased from Charles River Laboratories(Beijing, China) and housed in a room with a 12-h light/dark cycle, five rats per cage, at a temperature of 20–22 °C and relative humidity of 55–65%. Following a one-week adaptation period, 40 rats were randomly assigned to Control group, I/R group, I/R + SMYA group, and I/R + Aspirin group, with 10 rats in each group. For the I/R group, I/R + SMYA group, and I/R + Aspirin group rats, cardiac ischemia was induced under anesthesia by inhalation of 5% isoflurane followed by 2% isoflurane maintenance, and the rats were placed on the operating table. The laboratory animal ventilator was connected, and the surgical area was disinfected with iodophor. The rats were positioned at the 3rd/4th left rib space for heart exposure. Cardiac ischemia was simulated in the animals using a surgical suture (6.0 size) to ligate the proximal portion of the left anterior descending artery. The ligation was considered successful when the ligated part of the myocardium became clearly white. The ligature wire was loosened after 60 min to simulate reperfusion. After the closure of the chest, the rats were placed in clean enclosures and given appropriate post-surgical care. The rats were allowed to recover for one day before treatment. The rats in the I/R + SMYA group were orally administered 8.64 g/kg/d SMYA, while those in the I/R + Aspirin group received 50 mg/kg/d Aspirin. The rats in the other groups received an equivalent volume of saline. The experimental protocols ensured the well-being of the rats.

### Echocardiography

On day 28, echocardiography was performed on all rats in each group to evaluate cardiac function. A high-resolution ultrasound imager, Vevo 3100 (VisualSonics, Canada) MX250 probe, was used to examine the cardiac function of the rats at 21 Hz. The rats were placed on thermal blankets at 37 °C after shaving their chest hair and were anesthetized by inhaling 2% isoflurane. To obtain images of the papillary muscle in a horizontal short-axis ultrasound and M-ultrasound, the probe was rotated 90°. Images were taken to determine the values of left ventricular fractional shortening (LVFS), left ventricular ejection fraction (LVEF), left ventricular end-systolic diameter (LVEDs), and left ventricular end-diastolic diameter (LVEDd). Three consecutive cardiac cycles were measured, and the average of the three measurements was taken.

### H&E staining

To evaluate tissue damage in the colon and myocardium, H&E staining was conducted. Following deep anesthesia with 5% isoflurane, tissues were harvested after euthanasia of the rats. Myocardial tissues were collected from the I/R region, while colonic tissues were washed in phosphate-buffered saline (PBS). Tissues were fixed in 4% paraformaldehyde for 48 h, embedded in paraffin, and sliced into 4 µm sections. These sections were then dewaxed and rehydrated by immersing them in xylene and gradient ethanol, and subsequently stained with hematoxylin for 5 min and eosin for 1 min. Finally, neutral gum was used to seal the sections, and the tissue samples were observed under an optical microscope (Olympus, Tokyo, Japan).

### Gut microbiota analysis

In this study, fresh fecal samples were obtained from rats’ colons, transferred into sterile cryotubes, and frozen in liquid nitrogen. The 16S rDNA sequencing was carried out to study the microbial diversity of the fecal samples using Illumina-based deep sequencing, universal primers 338F: 5'-ACTCCTACGGGAGGCAGCA-3’ and 806R: 5'-GGACTACHVGGGTWTCTAAT-3 and the V3-V4 region of the 16S rRNA gene was amplified. The polymerase chain reaction (PCR) was conducted in a 20 µL reaction volume, consisting of 13.25 µL of H_2_O, 2.0 µL of 10 × PCR ExTaq Buffer, 2.0 µL dNTP, 1.0 µL of each primer at a concentration 10 mmol/L, 0.5 µL of 100 ng/mL template DNA, and 0.25 µL of 5 U/mL ExTaq. The PCR cycling conditions were 95 °C for 5 min, 30 cycles of 95 °C for 30 s, 58 °C for 20 s, and 72 °C for 6 s, with the final extension step at 72 °C for 7 min. This was followed by purification and recovery of the amplified products through 1% agarose gel electrophoresis. High-quality sequences were clustered/denoised using USEARCH at a similarity threshold of 97%. The species annotation at the genus and phylum levels was performed using RDP Classifier. The microbial diversity was assessed using α-diversity indices, including Chao1, ACE, Simpson’s and Shannon indices, and rarefaction curve with Motherv.1.30. The β-diversity analysis was performed using NMDS and PCoA at corresponding distances based on the distance matrix in R. Finally, statistically significant biomarkers were identified using LefSe analysis based on the LDA scores between groups.

### Enzyme-linked immunosorbent assay

In this research, after 28 days of treatment, rats were fasted for 8 h and anesthetized with 5% isoflurane inhalation to collect 5 mL of abdominal aorta blood. The collected blood was centrifuged at 1000 rpm and 4 °C for 10 min to separate the serum supernatant. A sample of colon tissue weighing 0.5 g was collected into a centrifuge tube and mixed with 1 mL of normal saline. The tissue was then homogenized using magnetic beads and a homogenizer, followed by centrifugation at 2500 rpm and 4 °C for 10 min. The supernatant of the tissue homogenate was collected for further analysis. An ELISA kit was used to assess the concentration of TNF-α, IL-6, IL-1β, IL-17A, and TLR4 in both the serum and colon tissue of the rats. The concentrations of LPS, CK-MB, and LDH in the serum were also measured using the same ELISA kit as per the manufacturer’s instructions.

### Western blot analysis

In this study, the BCA assay kit (Beyotime) was utilized to quantify the protein levels in the colon and heart tissues. To this end, the tissue samples were lysed using a lysis solution and homogenized on ice for 45 min. After centrifugation at 1000 rpm and 4 °C for 10 min, the supernatant was collected for further analysis. SDS-PAGE was performed using a 12% separating gel and a 5% stacking gel. Each well was loaded with 60 µg of total protein, electrophoresed, and transferred to a PVDF membrane. The membrane was incubated with TLR4 (1:1000), MyD88 (1:1000), P65 (1:1000), Occludin (1:1000), β-actin (1:20,000), p-P65 (1:1000), ZO-1 (1:1000), or Lamin B1 (1:5000) antibody after being blocked with 5% skimmed milk powder. The PVDF membrane was incubated with the antibody incubation solution for 12 h at 4 °C, washed, and then subjected to an HRP-conjugated goat anti-mouse secondary antibody (1:1000 dilutions) for 2 h at room temperature on a shaker. After adequate washing, images were captured using a Tanon automated chemiluminescence analyzer, and the Image J software was used to calculate the grayscale values. The internal reference proteins used were β-actin and Lamin B1. The grayscale values of the target protein and the internal reference protein were normalized, and the final results were presented using the normalized ratio.

### Statistical analysis

The data was processed using GraphPad Prism 7.0 and presented as mean ± SD. Student’s t-test was used for comparison between two groups, and One-way ANOVA was used for multiple group comparisons. The association of differential microflora with inflammatory biomarkers was evaluated using Spearman’s correlation test. Benjamini-Hochberg (BH) method was followed for calibration. *P < 0.05, **P < 0.01 vs. Control group, *#P* < 0.05, *##P* < 0.01 vs. I/R group.

## Results

### HPLC analysis of SMYA

**Fig. 1:**
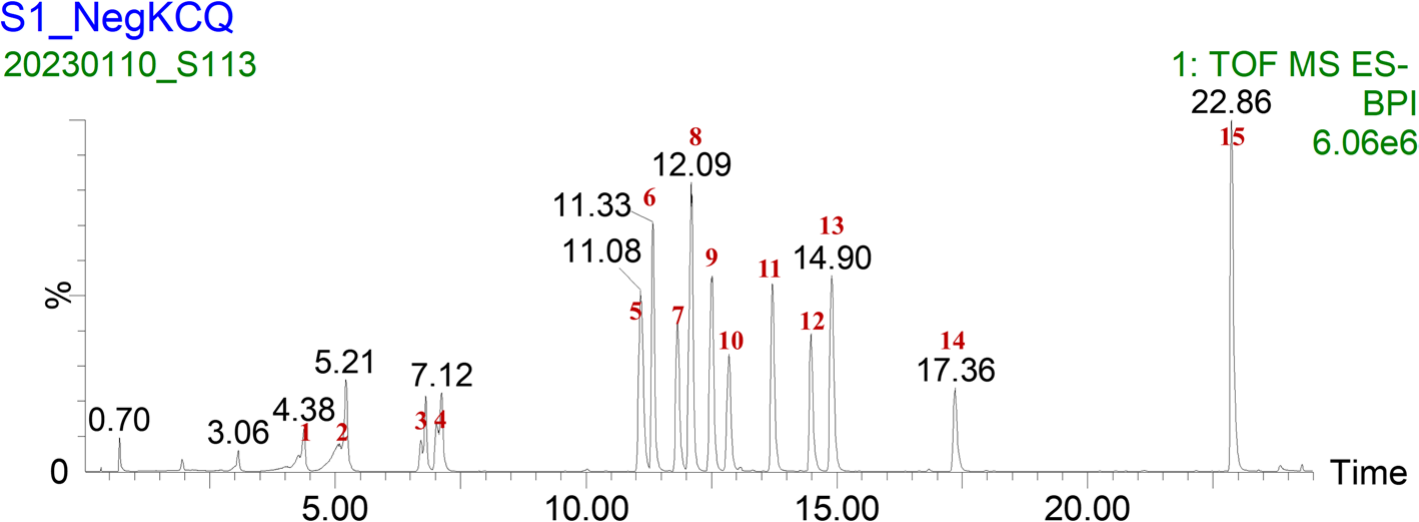
HPLC analysis of SMYA

HPLC analysis was used to identify the main components of SMYA. Fifteen chromatographic peaks were obtained based on the retention time consistency, as shown in Fig. 1. The identified compounds in SMYA are listed in Table 1.

**Table 1:** Determination results of compounds in SMYA

No.	Compounds name	Retention time(min)
1	Harpagide	4.38
2	Neochlorogenic acid	5.22
3	Chlorogenic acid	6.81
4	Cryptochlorogenic acid	7.13
5	Isoliquiritin	11.11
6	Rutin	11.33
7	Astragalin	11.85
8	Acteoside	12.11
9	Isochlorogenic acid C	12.53
10	Isochlorogenic acid A	12.87
11	Isochlorogenic acid B	13.74
12	Angoroside C	14.5
13	Liquiritin	14.9
14	Harpagoside	17.38
15	Glycyrrhizic acid	22.85

### Protective effect of SMYA on the myocardium

**Fig. 2:**
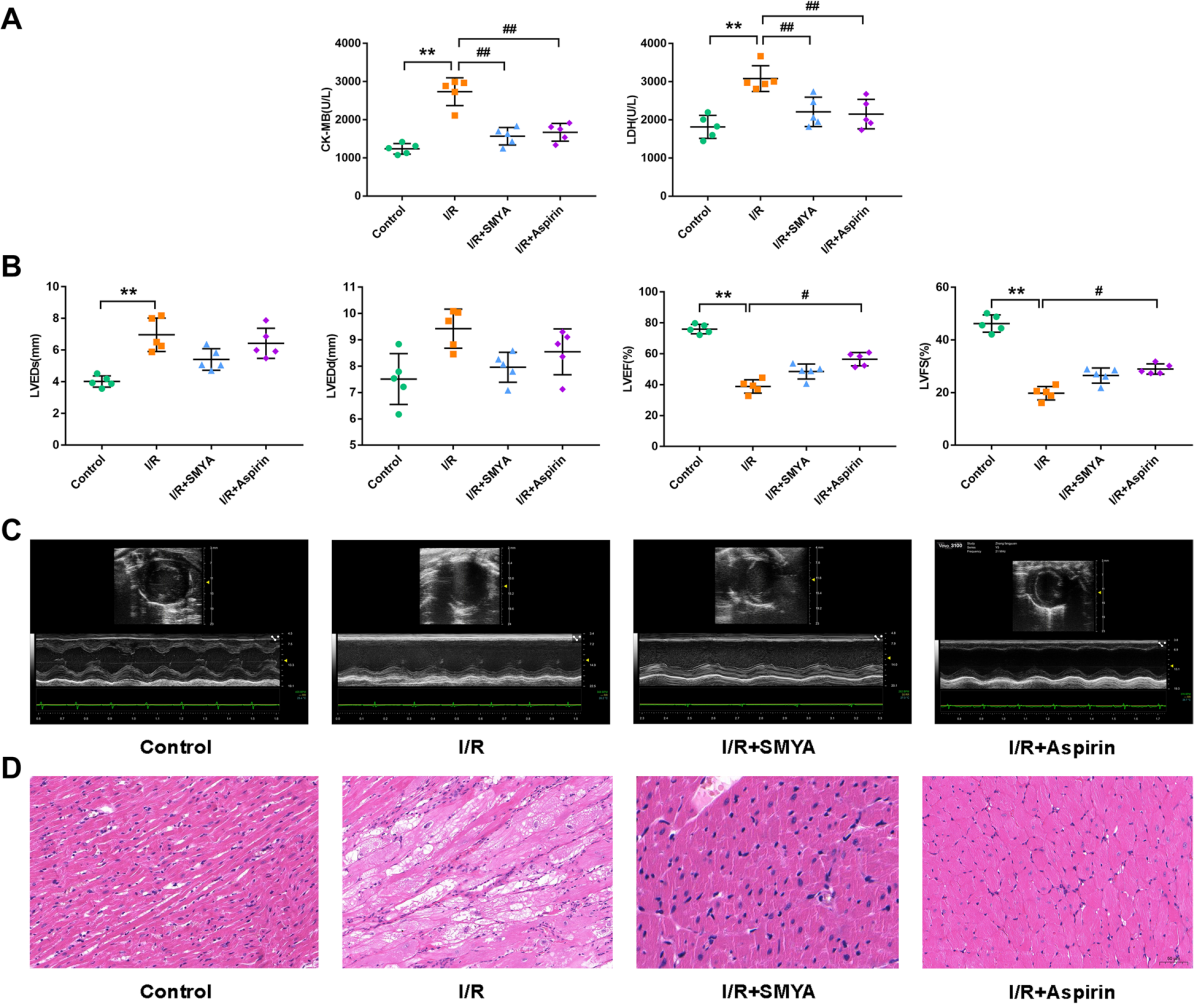
SMYA alleviates I/R-induced myocardial damage. **A** Markers of myocardial damage: CK-MB and LDH. **B** Echocardiography index levels of left ventricular end-systolic diameter (LVEDs), left ventricular end-diastolic diameter (LVEDd), left ventricular ejection fraction (LVEF), and left ventricular fractional shortening (LVES), measured by echocardiography. Data represented as Mean ± SD (*n* = 5). **C** Echocardiography of rat heart. **D** H&E staining of heart tissue in each group. **P < 0.01 vs. Control group*, #P* < 0.05 and *##P* < 0.01 vs. I/R group

To evaluate the efficacy of SMYA against myocardial damage, the serum levels of lactate dehydrogenase (LDH) and creatine kinase isoenzyme (CK-MB) were measured. Compared to the control group, the I/R group had significantly higher levels of CK-MB and LDH, while the administration of SMYA and Aspirin resulted in a significant decrease in these serum indices in I/R rats (Fig. 2A). Therefore, SMYA could effectively reduce serum CK-MB and LDH levels, thereby protecting the myocardium and reducing myocardial injury. Echocardiography was used to assess cardiac function. The treatment with SMYA led to a reduction in LVEDs and LVEDd values and an increase in LVEF and LVES values, although the difference was not statistically significant (Fig. 2B). The I/R group exhibited enlarged ventricles and thinner ventricular walls than the control group. Moreover, the heart tissue of the I/R group showed evident structural damage, while the administration of SMYA and Aspirin resulted in improved ventricular and ventricular wall structures in myocardial I/R rats (Fig. 2C). The myocardial cells’ structural changes were examined by analyzing H&E-stained cross-sections of the heart. The I/R rats displayed disordered myocardial filaments and varying degrees of necrosis. However, the administration of SMYA and Aspirin resulted in a significant reduction in myocardial tissue damage (Fig. 2D).

### SMYA reduces serum expression of inflammatory factors

**Fig. 3:**
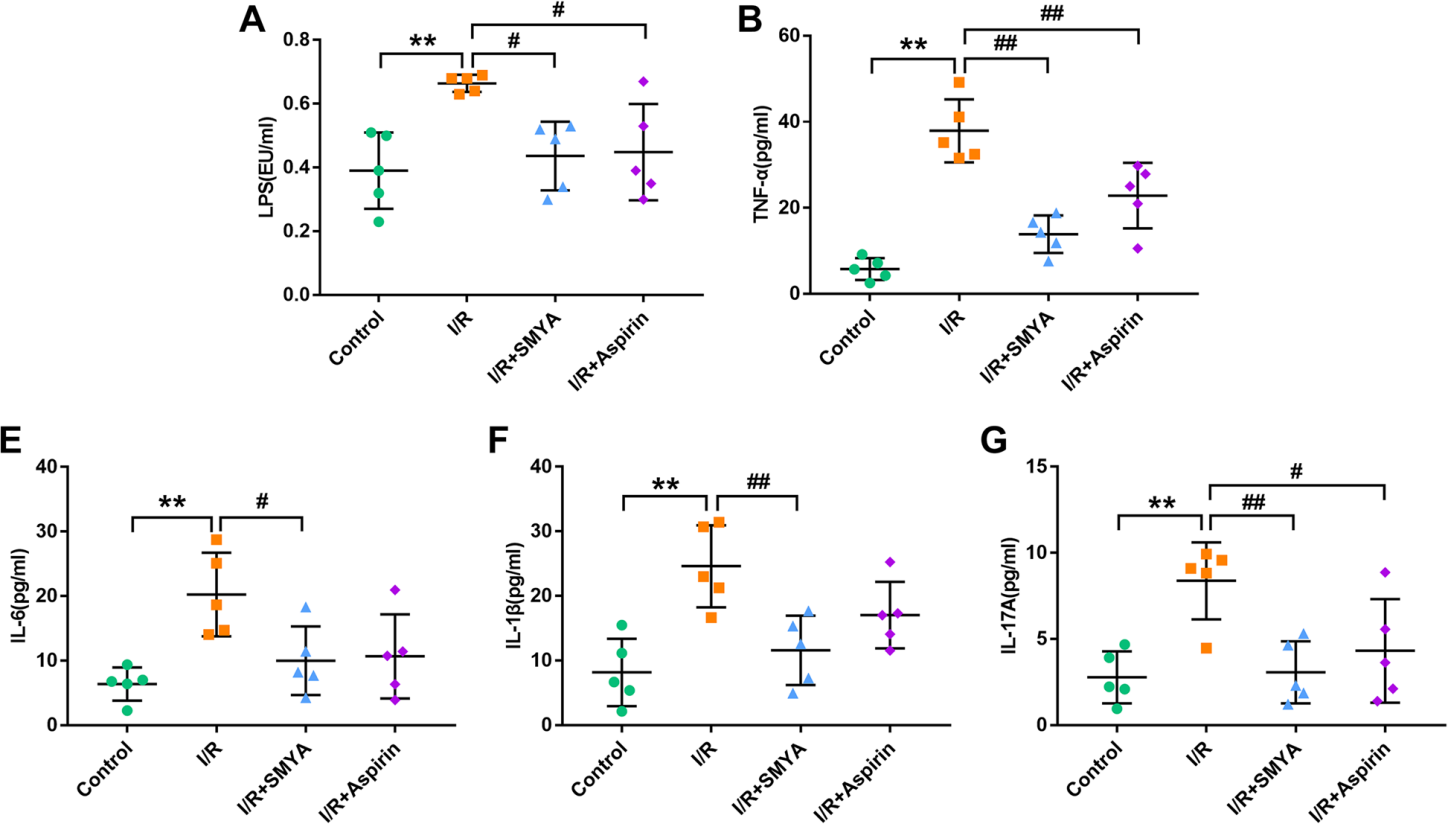
SMYA reduces serum expression of inflammatory factors. **A-G** LPS and serum inflammatory factors: TNF-α, IL-6, IL-β, and IL-17A. Data represented as Mean ± SD (*n* = 5). **P < 0.01 vs. Control group, *#P* < 0.05 and *##P* < 0.01 vs. I/R group

To investigate the effectiveness of SMYA in combating serum inflammation, various inflammatory markers were examined. The I/R group showed significantly higher levels of LPS compared to the control group, while the levels were reduced in the I/R + SMYA and I/R + Aspirin groups (Fig. 3A). The I/R group also exhibited a significant increase in downstream inflammatory factors (TNF-α, IL-6, IL-1β, and IL-17A). However, SMYA treatment led to a statistical reduction in these inflammatory cytokines (Fig. 3B-G). The I/R model demonstrated a notable elevation in serum LPS levels, which could enter the systemic circulation through the intestinal barrier, activate TLR4 signaling, and release downstream inflammatory factors. SMYA administration could effectively reduce LPS levels, the secretion of downstream inflammatory cytokines, and the level of inflammation in the serum.

### SMYA inhibits the expression of myocardial proteins in the TLR4/NF-κB pathway

**Fig. 4:**
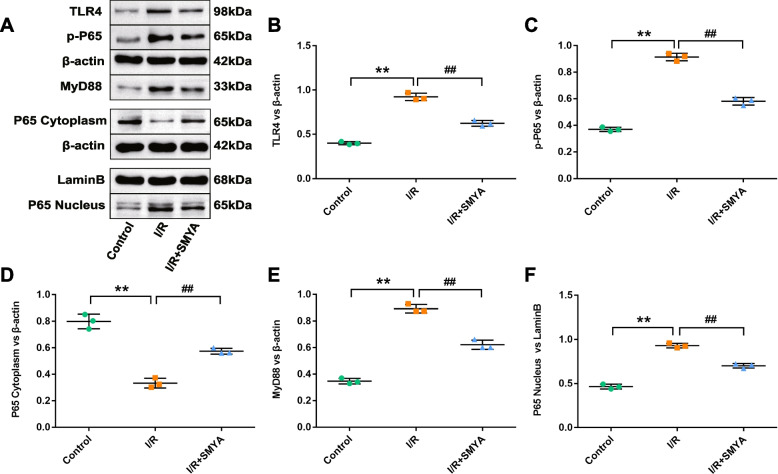
SMYA inhibits myocardial TLR4/NF-κB pathway. **A** Western blot method to detect protein level of TLR4, p-P65, MyD88, p65 cytoplasm, p65 nucleus heart tissue. **B-F** Comparison of myocardial protein expression levels between groups. Data represented as Mean ± SD (*n* = 3). **P < 0.01 vs. Control group, *##P* < 0.01 vs. I/R group

To investigate the mechanism of SMYA in reducing inflammation in an in vivo I/R model, the myocardial proteins in the TLR4/NF-κB pathway were analyzed (Fig. 4A). The discussion mainly focused on the SMYA mechanism; thus, the I/R + Aspirin group was no longer included. MyD88 and TLR4 proteins were significantly upregulated in the I/R group and downregulated after SMYA administration (Fig. 4B, C). In the I/R group, myocardial tissue p-P65 protein and p65 nuclear protein expression were upregulated, while in the I/R + SMYA group, the protein expression was considerably downregulated (Fig. 4D, E). Conversely, p65 cytoplasmic protein expression was downregulated in the I/R group and upregulated in the I/R + SMYA group (Fig. 4F). Therefore, SMYA can inhibit the expression of proteins in the TLR4/NF-κB pathway, reducing the release of inflammatory factors.

### Effects of SMYA on gut microbiota

**Fig. 5:**
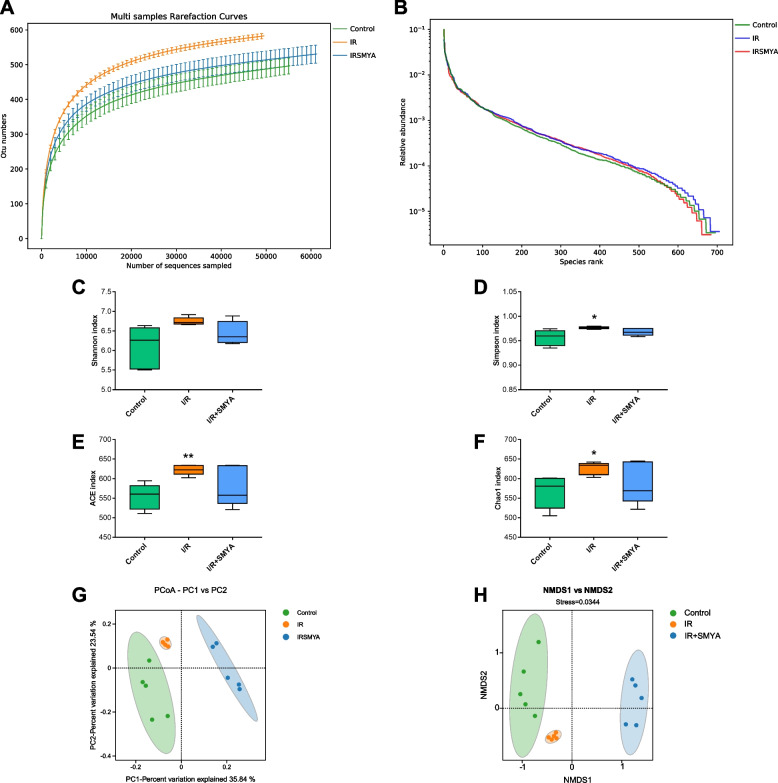
SMYA regulates gut microbiota. **A** Rarefaction Curve. **B** Rank Abundance Curve. **C-F** Shannon index, Simpson index, Chao1 index, and Ace index in α-diversity. **G** β-diversity PCoA analysis. **H** β-diversity NMDS analysis

In this study, 16S rDNA sequencing was performed on the colon feces of rats. The Rarefaction Curve analysis indicated that the sequencing data were adequate for data analysis, as the curves for the three groups were flat. However, the I/R group showed a higher alpha-diversity index compared to the other groups, which was also supported by the Rank Abundance Curve (Fig. 5A, B). Although there was no significant difference in the Shannon index among the three groups, Simpson’s index showed a significant difference between the I/R and control rats, indicating a higher diversity in the I/R group. The species abundance and evenness were also higher in the I/R group rats, indicating a higher diversity. Additionally, the Ace and Chao1 indices showed a more significant number of species in the I/R rats compared to the control rats (Fig. 5C-F). The alpha diversity of the I/R + SMYA group decreased, although the difference was not statistically significant when compared to the I/R group, and the trend was similar to that observed in the normal group. For beta diversity, similarity analysis among the three groups was conducted using principal coordinate analysis (PCoA) and non-metric multi-dimensional scaling (NMDS). PCoA classified the samples based on their main elements and structure, assuming linear relationships in the data, whereas NMDS differentiated the samples based on the non-linear structure of ecological data. Both methods could distinguish the three groups, with an R-value of 0.91 and a *p*-value of 0.001, and a Stress value of 0.0034 in NMDS, indicating better clustering and separation of the groups (Fig. 5G, H). These results suggest a significant change in gut microbiota in the I/R and I/R + SMYA groups.

**Fig. 6:**
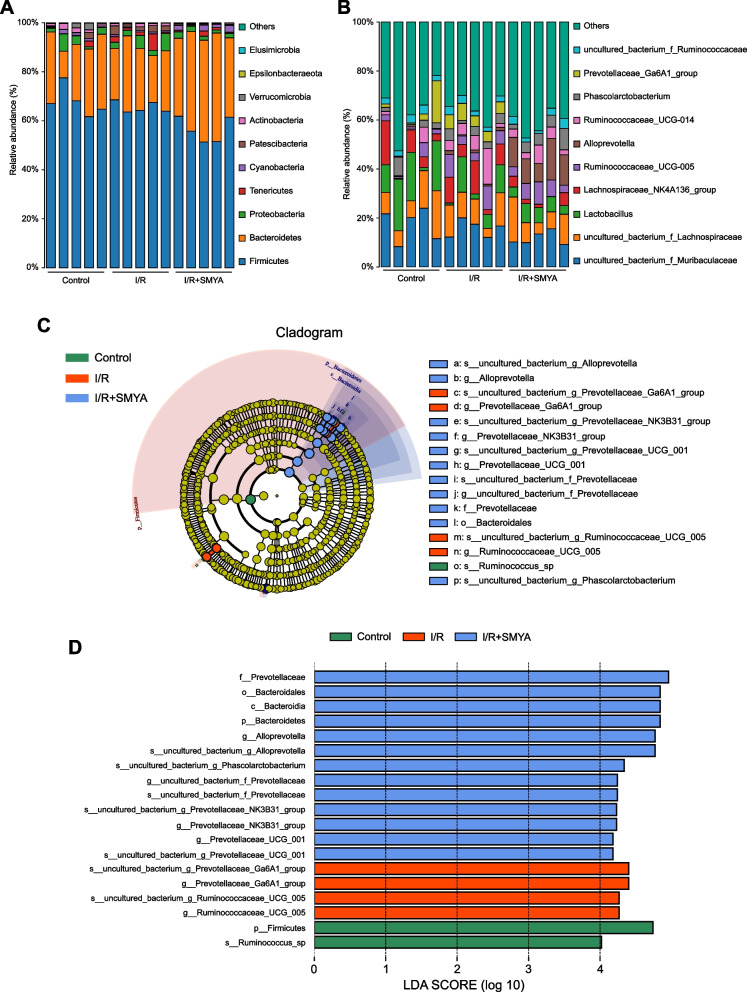
Analysis of gut microbiota composition and differences among groups. **A** Species histogram per sample at the phylum level. **B** Species histogram per sample at the genus level. **C** Cladogram for Lefse analysis. **D** LAD value distribution histogram

The analysis of gut microbiota in the I/R rats revealed an increase in Tenericutes and Patescibacteria at the species level and a decrease in Cyanobacteria at the phylum level. However, in the I/R + SMYA rats, these changes were reversed, and there was an increase in Bacteroidetes (Fig. 6A). Previous studies have reported an increase in Firmicutes and a decrease in Bacteroidetes in patients with CAD. Although the I/R rats did not show such a trend, the administration of SMYA resulted in a significant reduction in Firmicutes and an increase in Bacteroidetes. In the I/R group, there was an elevation at the genus level for Ruminococcaceae_UCG-014, Ruminococcaceae_UCG-005, and Lachnospiraceae_NK4A136. However, the administration of SMYA reduced this elevation and increased Phascolarctobacterium and Alloprevotella (Fig. 6B). Linear Discriminant Analysis Effect Size (LefSe) assessment of the differential bacteria in the three groups showed significantly enriched LDA scores (LDA > 4), with an increase in Prevotellaceae_Ga6A1_group (genus, species) and Ruminococcaceae_UCG_005 (genus, species) in the I/R group. However, SMYA inhibited these bacteria by increasing Bacteroidetes (phylum, class, order), f__Prevotellaceae (family, genus, species), Prevotellaceae_UCG_001 (genus, species), g__Prevotellaceae_NK3B31_group (genus, species), g__Alloprevotella (genus, species), s__uncultured_bacterium_g_Phascolarctobacterium (genus) (Fig. 6C, D). In conclusion, SMYA could regulate gut microbiota structure in myocardial I/R rats.

### Correlation analysis of gut microbiota and serum inflammatory factors

**Fig. 7:**
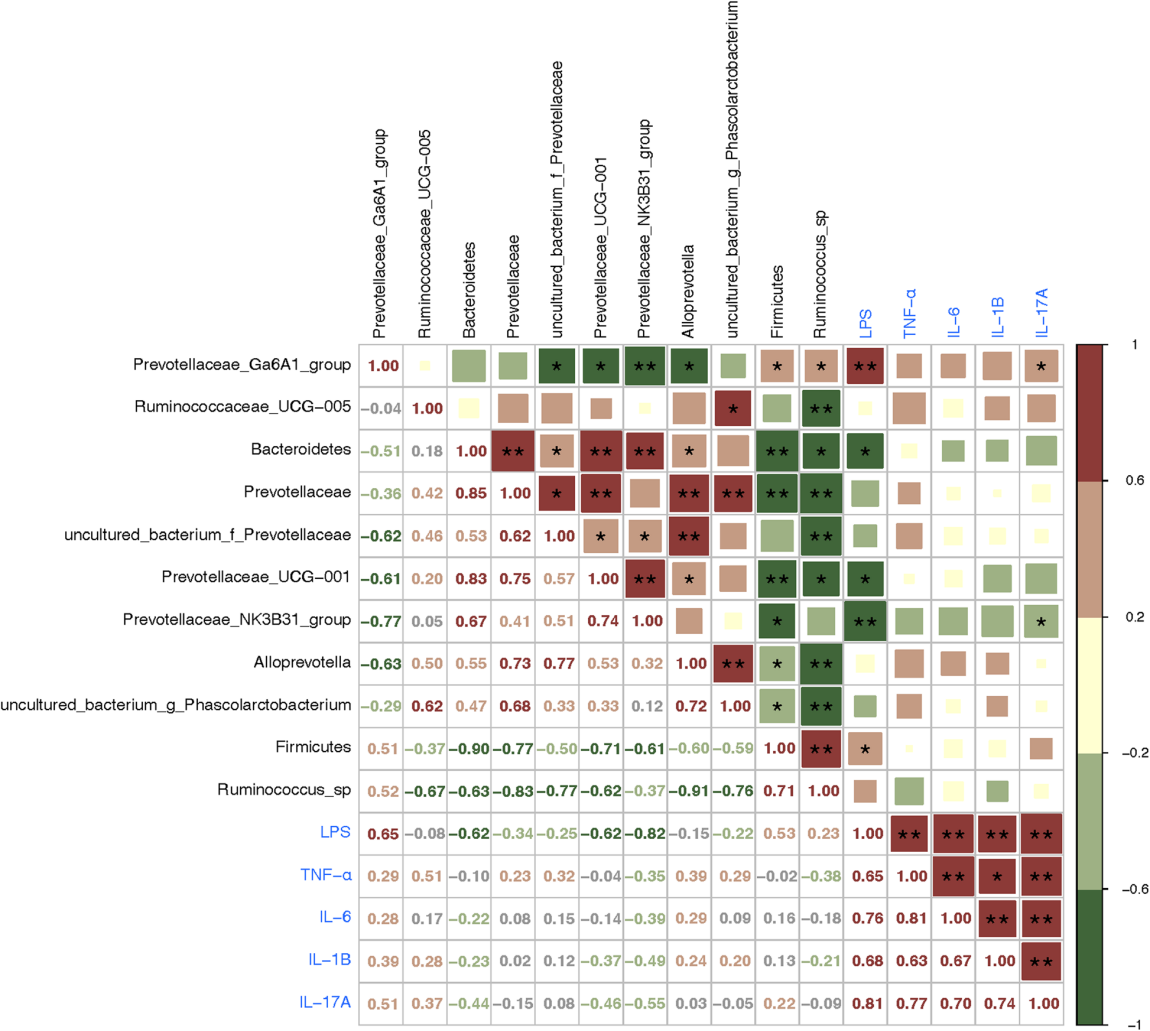
Correlation analysis between differential microbiota and inflammatory factors. Spearman’s correlation between differential microbiota, and serum inflammatory factors. The black color represents gut microbiota, the blue color represents the serum inflammatory factor. Data represented as Mean ± SD (*n* = 5). Correlation coefficient values in the lower left panel and the upper right are BH-corrected *P < 0.05, **P < 0.01

The study analyzed the correlation between the identified differential microbiota and serum inflammatory factors. After removing evolutionary duplicate microbiota, eleven differential microbiota were analyzed using hierarchical clustering, Spearman’s correlation analysis, and subsequent correction using the BH method. The analysis revealed two correlation clusters between the serum LPS and inflammatory factors (Fig. 7). Bacteroidetes were found to be positively correlated with Prevotellaceae, Prevotellaceae_UCG_001, and Prevotellaceae_NK3B31 (adjusted P < 0.01). On the other hand, Firmicutes showed a positive correlation with Ruminococcus and Gram-positive bacteria and a negative correlation with most other differential bacteria, especially Bacteroidetes, Prevotellaceae, and Prevotellaceae_UCG_001 (adjusted *P* < 0.01). The serum LPS was positively correlated with Prevotellaceae_Ga6A1 and negatively correlated with Bacteroidetes, Prevotellaceae_UCG_001 (adjusted *P* < 0.05), and Prevotellaceae_NK3B31 (adjusted *P* < 0.01). Additionally, serum IL-17A was positively correlated with Prevotellaceae_Ga6A1 and negatively correlated with Prevotellaceae_NK3B31. Interestingly, the increase in individual microbiota in the I/R + SMYA group showed a negative correlation with the inflammatory factors, whereas the increased microbiota in the I/R group showed a more positive correlation. These findings suggest a potential association between gut microbiota, LPS, and inflammatory factors.

### SMYA protects the intestinal barrier and inhibits inflammatory expression in colon tissue

**Fig. 8:**
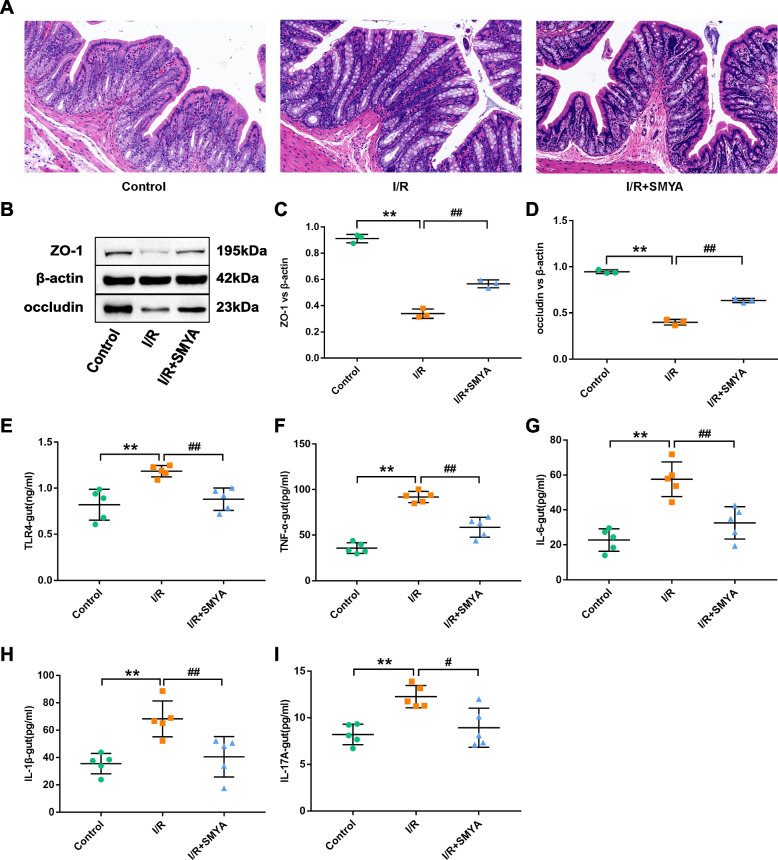
Effects of SMYA on colon tissue. **A** H&E staining of colonic tissue in each group. **B** Western blot method to determine ZO-1, occludin protein levels in colon tissue. **C**, **D** Comparison of protein level expression between groups. Data represented as Mean ± SD (*n* = 3). **E-I** The levels of TLR4, TNF-α, IL-6, IL-β, and IL-17A in colon tissue homogenates. Data represented as Mean ± SD (*n* = 5). **P < 0.01 vs. Control group, *##P* < 0.01 vs. I/R group

To validate the effect of SMYA on the gut mucosa, H&E staining was performed. The I/R rats showed mucosal damage, altered villi structure, loss of crypt cells, and inflammatory cell infiltration that could extend up to the mucosal muscle layer, in contrast to the control rats (Fig. 8A). However, the I/R + SMYA group exhibited improved colonic mucosal damage, reduced inflammatory infiltration, and a better villi structure than the I/R group. The I/R rats also showed significantly decreased expression of ZO-1 and occludin tight junction proteins compared to the control rats, while the I/R + SMYA group exhibited higher levels of these proteins and a weakened intestinal permeability (Fig. 8B-D). These results suggest that SMYA can reduce intestinal mucosal damage, maintain the intestinal barrier, and decrease intestinal permeability. Moreover, the study explored the effect of SMYA on colonic inflammation by examining the colonic tissue homogenate levels of TLR4 and its downstream inflammatory factors (IL-6, IL-1β, TNF-α, and IL-17) biomarkers. The I/R rats showed significantly higher levels of these markers than the control rats, whereas the I/R + SMYA group exhibited lower cytokine expressions. These findings indicate that SMYA has an inhibitory effect on TLR4 levels and its downstream inflammatory factors in colonic tissues (Fig. 8E-I).

## Discussion

Myocardial injury can trigger an inflammatory response that persists throughout the stages of CAD, resulting in the release of inflammatory cytokines such as IL-6 and TNF-α, which can cause damage to the heart and its components, including cardiomyocytes and endothelial cells, by recruiting other inflammatory cells [27]. In addition, persistent low-grade inflammation is associated with coronary atherosclerosis, and patients receiving anti-inflammatory therapy have been found to have an improved cardiovascular prognosis [28]. Several studies have demonstrated the anti-inflammatory properties of SMYA, including its ability to inhibit cardiac hypertrophy and fibrosis, reduce lipid deposition [29], decrease atherosclerotic plaque area, and stabilize fragile AS plaque [30]. Additionally, a study found that Jia-Wei-Si-Miao-Yong-An decoction can regulate intestinal flora [31]. Despite these findings, no previous studies have investigated the regulation of gut microbiota by SMYA or explored the link between the myocardial improvement effects of SMYA and inflammation and gut microbiota. Therefore, we conducted a study to investigate these relationships.

In the current research, SMYA was found to have a positive impact on alleviating myocardial function, although the increase in LVEF and LVES values was not as significant as aspirin. Moreover, SMYA was observed to improve myocardial injury by reducing serum CK-MB and LDH levels. Additionally, the study found that SMYA significantly reduced serum levels of inflammatory factors, namely TNF-α, IL-6, IL-1β, and IL-17, which are elevated in myocardial I/R rats. This indicates that SMYA has the potential to reduce circulating inflammation levels and improve CAD by inhibiting pathways associated with inflammation.

TLR4 is an essential member of the Toll-like receptor family that plays a crucial role in the development and rupture of atherosclerotic plaques. Activation of TLR4 triggers the TLR4/NF-κB signaling cascade, promoting the expression of downstream inflammatory biomarkers (IL-6 and TNF-α), vascular endothelial injury, smooth muscle proliferation, and other responses. Moreover, the NF-κB pathway activation is crucial for the treatment of CAD [32]. The study found that the expression of myocardial proteins, namely TLR4, MyD88, and p-P65, and downstream inflammatory factors were observed in myocardial I/R rats, consistent with previous reports [30, 33]. After SMYA treatment, these factors were significantly reduced, indicating the inhibitory effect of SMYA on TLR4/NF-κB signaling.

One of the main triggers of TLR4/NF-κB signaling is the activation of TLR4 signaling induced by LPS. LPS, which is a major component in the cytoderm of Gram-negative bacteria, also plays a critical role in chronic low-grade inflammation. Gut microbiota is the primary source of LPS in humans [8], and lower levels of LPS can lead to low-grade inflammation, raised cholesterol levels, and atherosclerotic plaque formation [34]. LPS from *Escherichia coli* localizes in atherosclerotic plaques and interacts with TLR4, triggering inflammation and activating macrophages [35]. After TLR4 recognizes LPS infection, it activates intracellular tail-like structures, recruits myeloid differentiation factor MyD88, activates NF-κB, and further releases pro-inflammatory cells [36, 37]. In the present study, a prominent rise in serum LPS was observed in rats with myocardial I/R, and the number of LPS entering the systemic circulation increased. Previous studies have reported that LPS enters the systemic circulation and causes an inflammatory response involved in the development of cardiovascular events; thus, it can serve as a biomarker of CAD [38, 39]SMYA was found to lower the expression of LPS and inhibit TLR4/NF-κB signaling, reducing the expression of downstream inflammatory factors and circulating levels of inflammation.

To investigate the interaction between gut microbiota and inflammation in myocardial I/R rats and the effect of SMYA on gut microbiota, 16S rDNA sequencing was performed. The diversity in the I/R group increased, consistent with previous studies [14, 40]. According to the β-diversity analysis, the 3 groups could be better separated with significant changes. The phylum levels were dominated by Firmicutes and Bacteroidetes. Although no significant change was observed in the F/B ratio of myocardial I/R rats, a pronounced decline in the F/B ratio was noted in the SMYA group. A lower F/B ratio is beneficial for improving inflammation [41]. Patients with CAD have been reported to exhibit a significant decrease in Bacteroidetes, in contrast to healthy individuals, and an increase in the proportion of Firmicutes [42]. SMYA reversed this trend by significantly increasing the proportion of Bacteroidetes and decreasing Firmicutes. A moderate increase in Bacteroidetes is beneficial for CAD, as it reduces lipopolysaccharides and inhibits inflammatory responses [43]. Furthermore, Tenericutes and Patescibacteria [44] expression in the gut microbiota of patients with CAD has been reported to increase, which is consistent with the current findings.

The richness values of Ruminococcaceae_UCG-014 and Ruminococcaceae_UCG-005 at the genus level were slightly increased in the myocardial I/R group, similar to the trend observed between the I/R + SMYA and control groups. The proportion of Lachnospiraceae NK4A136 decreased in the I/R + SMYA group. A study has found that Resveratrol can lower the proportion of Lachnospiraceae_NK4A136 in mice consuming a high-fat diet and decrease the level of inflammation [45], indicating that SMYA may have a similar effect. Moreover, previous research has shown that an increased proportion of Phascolarctobacterium can enhance antioxidant effects in patients with CAD [46], and Alloprevotella can produce butyric acid, which is beneficial bacteria [47]. The I/R + SMYA group exhibited an increased proportion of Phascolarctobacterium and Alloprevotella. These preliminary findings suggest that SMYA can modulate the structure and ratio of gut microbiota in myocardial I/R rats.

To further explain the association between gut microbiota and inflammatory biomarkers in myocardial I/R rats, a correlation analysis was conducted between the major differential microbiota and serum inflammatory markers. Prevotellaceae_Ga6A1 was enriched in the gut microbiota of the I/R group and positively correlated with serum LPS and IL-17A, indicating that Prevotellaceae_Ga6A1 is associated with serum inflammatory factors. Previous research has suggested the involvement of gut inflammation and LPS in the regulatory mechanism of Prevotellaceae_Ga6A1 [48]. The Prevotellaceae_NK3B31_group was enriched in the I/R + SMYA group and showed a negative association with LPS and IL-17A expression. Prevotellaceae_NK3B31_group acts as a protective microbiota by producing butyric acid [49, 50], and the abundance of Prevotellaceae_NK3B31_group can be increased by using Berberine. SMYA had a similar effect, suggesting that it might modulate Prevotellaceae_NK3B31_group to suppress the expression of related inflammatory factors. LPS was negatively correlated with Bacteroidetes and Prevotellaceae_UCG_001. Bacteroidetes and Prevotellaceae_UCG_001 were enriched in the I/R + SMYA group, indicating that the more these bacteria express, the less LPS is expressed. Furthermore, other inflammatory factors were more positively correlated with Ruminococcaceae_UCG_005 and Prevotellaceae_Ga6A1_group and more negatively correlated with Bacteroidetes, Prevotellaceae_NK3B31_group, and Prevotellaceae_UCG_001. Although these trends were not statistically significant, they still suggest a relationship between differential microflora and serum inflammation. The changes in gut microflora might affect inflammatory factors, and SMYA might alleviate myocardial I/R injury by modulating anti-inflammatory microfloral richness, reducing pro-inflammatory bacteria and LPS production, and decreasing inflammation in vivo.

Research has demonstrated a link between inflammation and microbiota. Consequently, investigations have been conducted to examine the impact of gut microbiota on inflammatory factors and the colon. In the myocardial I/R group, the colon exhibited pathological alterations such as changes in intestinal villus structure, infiltration of inflammatory cells, and other forms of mucosal damage. However, SMYA was able to alleviate intestinal injury. According to various studies, LPS has the potential to elevate intestinal permeability, allowing it to enter the circulation and induce inflammation which can lead to subsequent cardiovascular attacks [14]. The equilibrium of intestinal microflora is significantly associated with the intestinal barrier, and any changes to the microflora can potentially harm the gut mucosal barrier, causing increased intestinal permeability. This heightened permeability, as observed in both patients with cardiovascular disease (CVD) and mice models, can subsequently increase the systemic inflammatory response. This, in turn, serves as a predictor of adverse cardiovascular outcomes [51]. Studies have indicated that damage to the gut barrier is associated with myocardial ischemic stress. Moreover, LVEF exhibits a positive correlation with mesenteric artery blood flow. Conversely, damage to the intestinal barrier and increased markers of intestinal permeability demonstrate a negative correlation with LVEF. Additionally, heightened intestinal permeability shows a negative correlation with mesenteric artery blood flow [14]. Thus, there is a potential for reduced blood flow in the mesenteric artery and heightened intestinal permeability in myocardial I/R. Our study measured intestinal permeability, and the results revealed a significant decrease in the expression of colonic proteins ZO-1 and occludin in the I/R group. However, after SMYA intervention, there was an increase in tight junction protein expression and a reduction in intestinal permeability. Therefore, SMYA exerts a protective effect on the intestinal mucosal barrier in myocardial I/R rats. Disruption of the intestinal barrier can lead to the translocation of gut microbiota and LPS, triggering the release of inflammatory factors. Inflammatory factors were markedly upregulated in the colon tissue homogenate in the I/R group, increasing the likelihood that these factors could penetrate the intestinal barrier and affect the heart. However, SMYA administration reduced the expression of colonic inflammatory factors, protected the intestinal tissues, maintained the intestinal barrier, and mitigated further heart damage caused by inflammation.

## Conclusions

In conclusion, our study revealed that SMYA could provide protection to the myocardium, reduce inflammation, and modulate gut microbiota in myocardial I/R rats. The mechanism of action possibly involves the modulation of gut microbiota and the safeguarding of the intestinal barrier to decrease the translocation of LPS and inflammatory factors. Additionally, it might involve the inhibition of the TLR4/NF-κB signaling pathway to mitigate myocardial damage caused by inflammatory factors. Our findings offer a novel scientific rationale for utilizing SMYA in the treatment of CAD.

## Supplementary Information

**Additional file 1.**

## Data Availability

Data are available from the corresponding author on reasonable request. The sequencing data used in this study are stored in NCBI SRA database (PRJNA940314). https://www.ncbi.nlm.nih.gov/sra/PRJNA940314.

## Abbreviations

CAD: Coronary artery disease
I/R: Ischemia/reperfusion
LPS: Lipopolysaccharide
TLR4: Toll-like receptor 4
MyD88: Myeloid differentiation factor 88
NF-κB: Nuclear factor-κB
p-P65: Phosphorylation p65
LVEDs: Left ventricular end-systolic diameter
LVEDd: Left ventricular end-diastolic diameter
LVEF: Left ventricular ejection fraction
LVES: Left ventricular fractional shortening
TNF-α: Tumor necrosis factor-α
IL-: Interleukin-
