# Correction: Effects of Si-Miao-Yong-An decoction on myocardial I/R rats by regulating gut microbiota to inhibit LPS-induced TLR4/NF-κB signaling pathway

**DOI:** 10.1186/s12906-023-04057-x

**Published:** 2023-07-01

**Authors:** Yuting Cui, Fangyuan Zhang, Weiming Xu, Ziyun Li, Jiaxi Zou, Ping Gao, Jingqing Hu

**Affiliations:** 1 Changchun University of Chinese Medicine, Changchun, China; 2 Institute of Basic Theory for Chinese Medicine, China Academy of Chinese Medical Sciences, Beijing, China; 3 China Science and Technology Development Center for Chinese Medicine, Beijing, China; 4 The First Afliated Hospital of Henan University of CM, Zhengzhou, China; 5 School of Acupuncture and Tuina, School of Regimen and Rehabilitation, Nanjing University of Chinese Medicine, Nanjing, China; 6 School of Basic Medical Sciences, Chengdu University of Traditional Chinese Medicine, Chengdu, China; 7 Afliated Hospital of Integrated Traditional Chinese and Western Medicine, Nanjing University of Chinese Medicine, Nanjing, China

**Correction: BMC Complement Med Ther 23, 180 (2023)**

**https://doi.org/10.1186/s12906-023-04013-9**

Following publication of the original article [1], the authors identified an error in Fig. 8D. The picture image was incorrect. The correct Fig. 8D is given below.
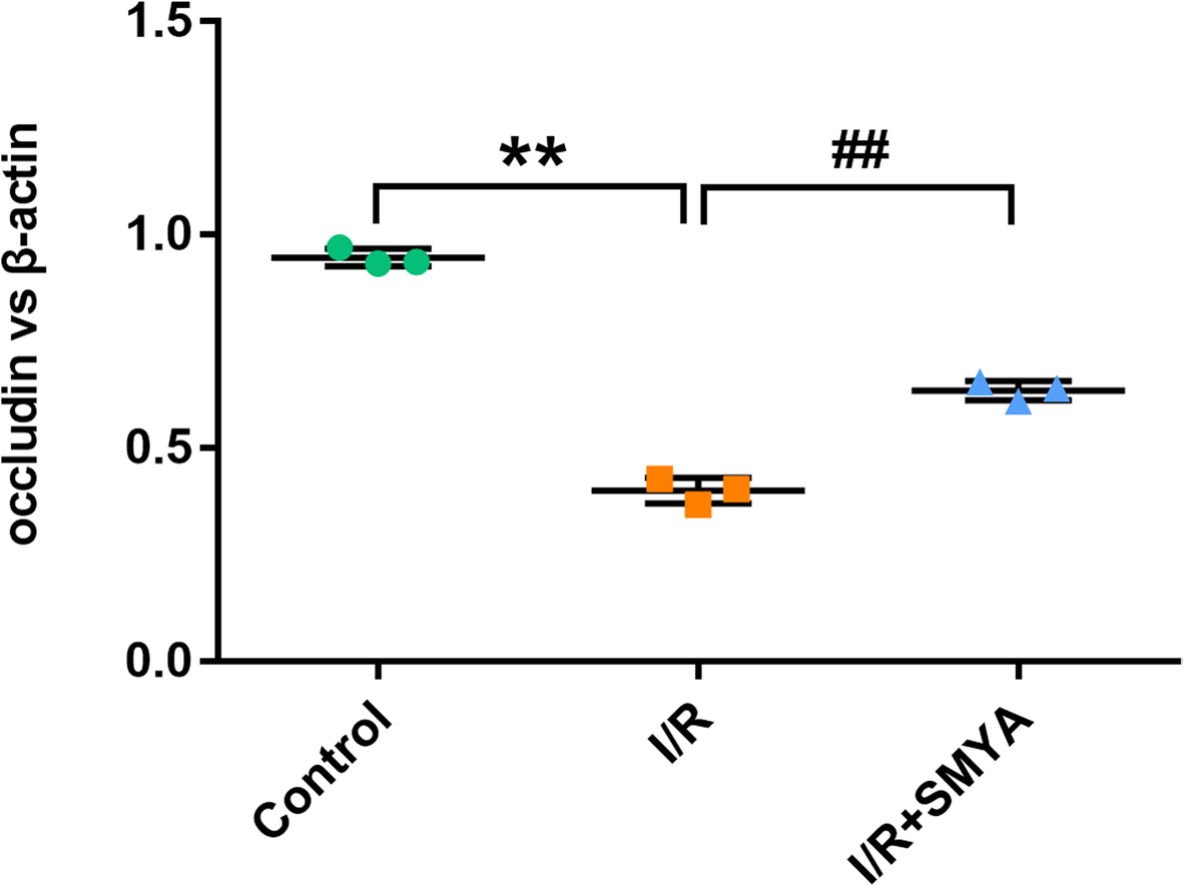


The original article has been corrected.
