# Supplementary material for: Effects of Si-Miao-Yong-An decoction on myocardial I/R rats by regulating gut microbiota to inhibit LPS-induced TLR4/NF-κB signaling pathway
Source: BMC Complement Med Ther. 2023 Jun 2;23:180. doi: 10.1186/s12906-023-04013-9 (PMC10236840; doi:10.1186/s12906-023-04013-9)

Figure 1

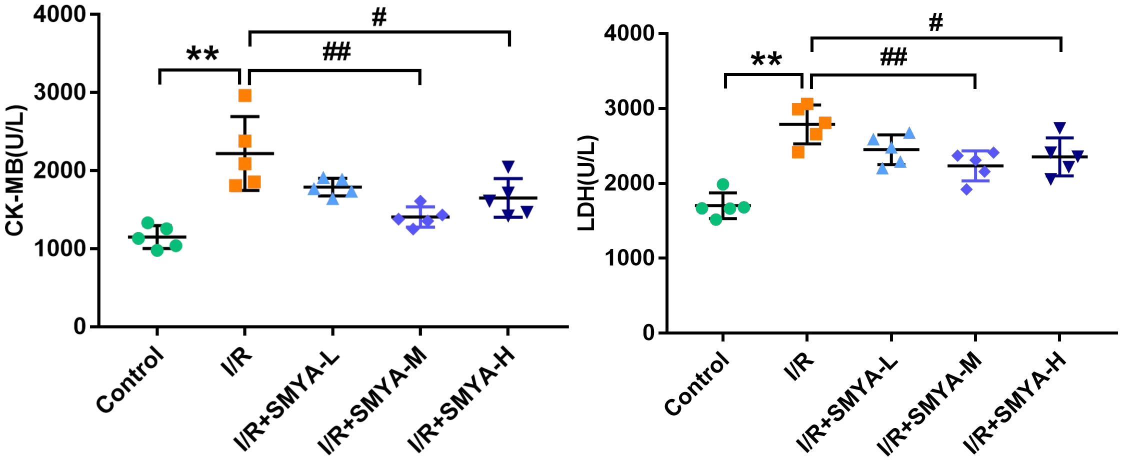

Figure 1 Markers of myocardial damage: CK-MB and LDH. **P<0.01 vs. Control group, #P<0.05 and ##P<0.01 vs. I/R group.

Western blot uncut blots

1. TLR4

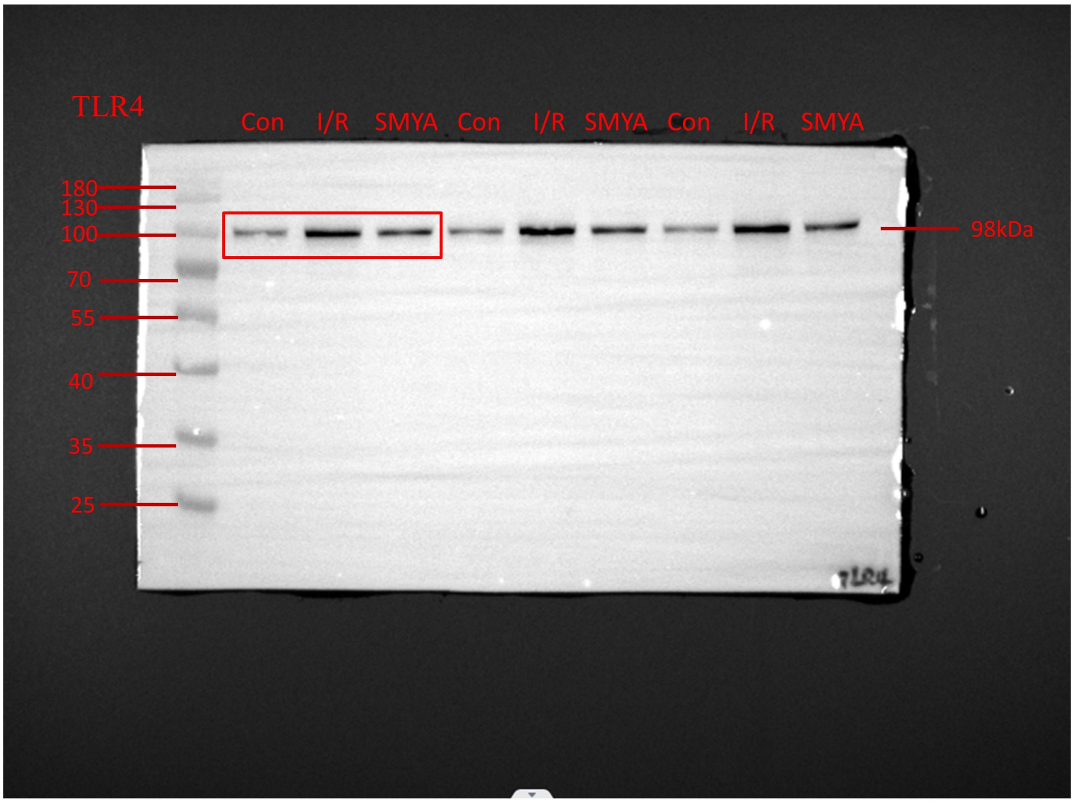

2. P-P65

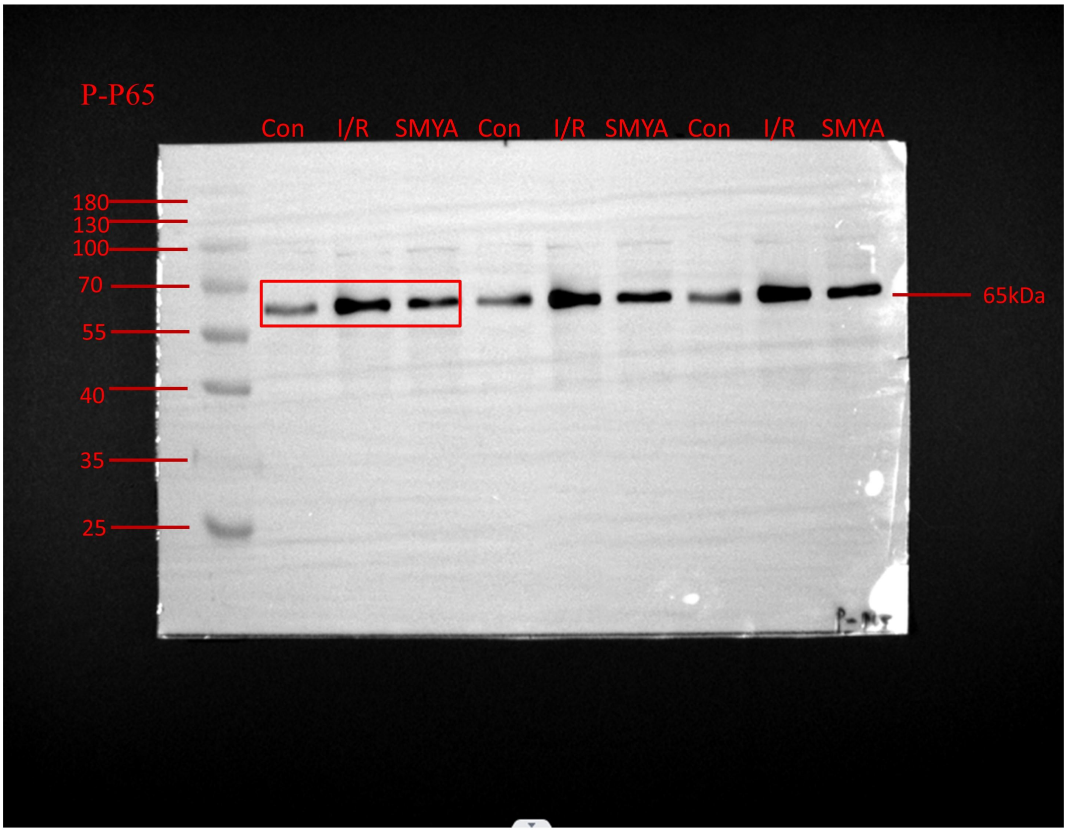

3. β-actin 1

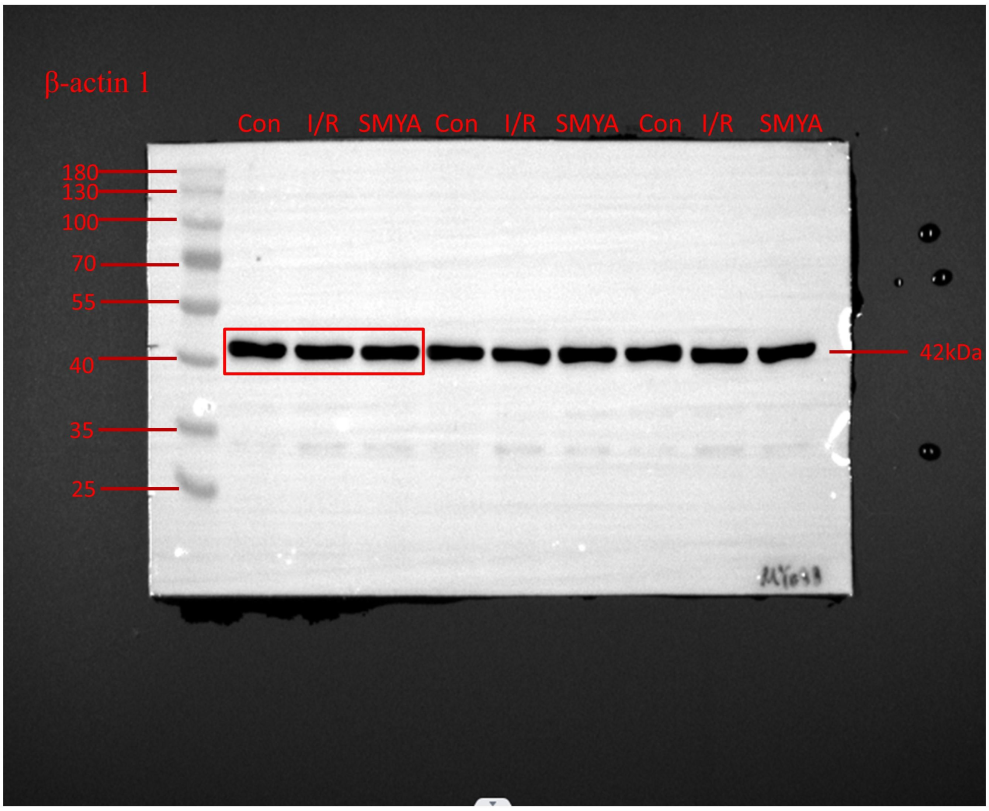

4. MyD88

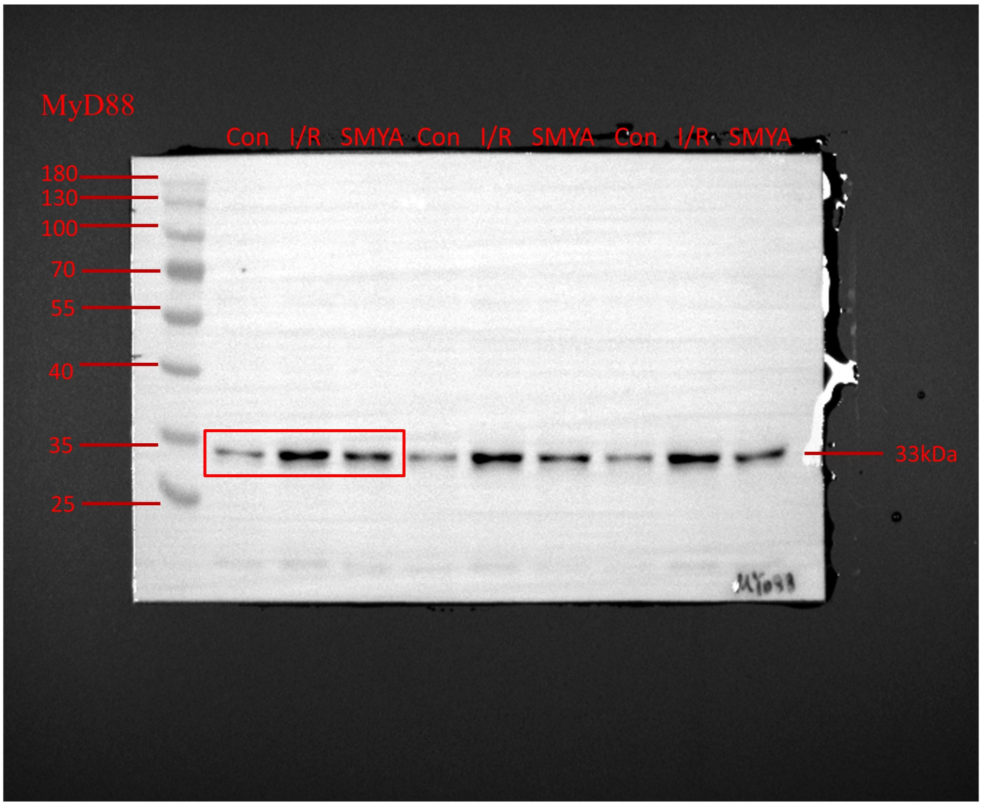

5. P65 Cytoplasm

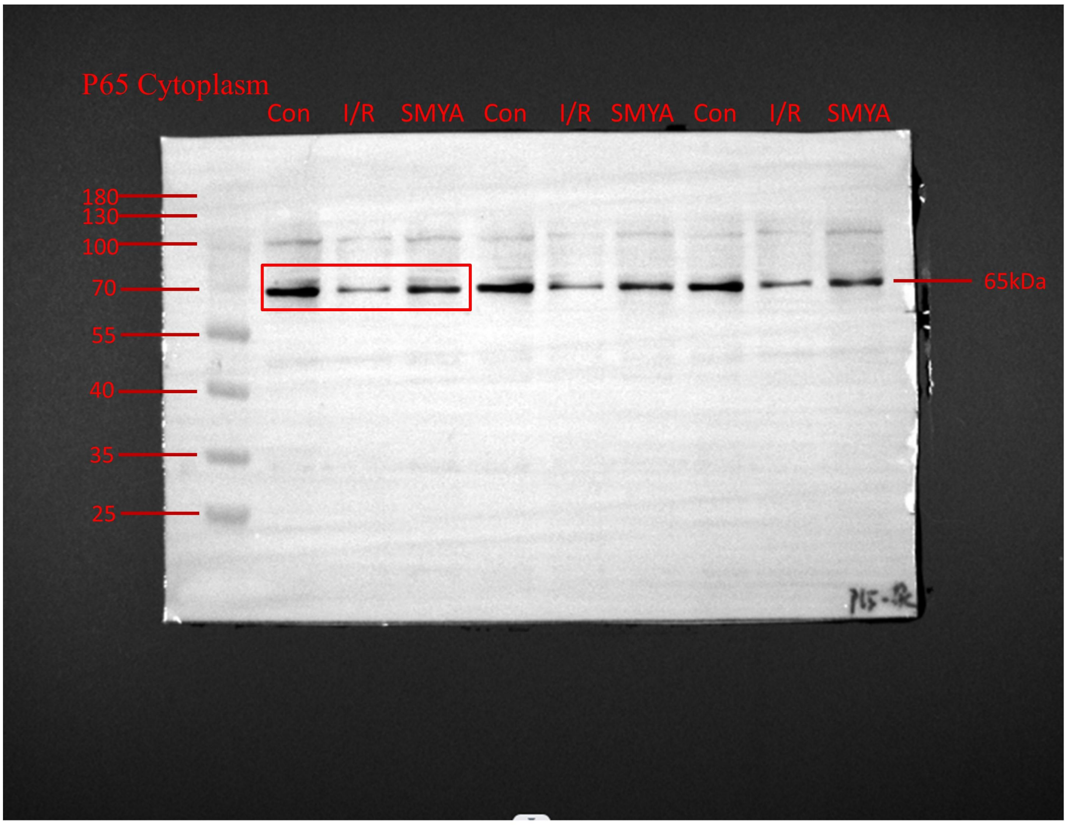

6. β-actin 2

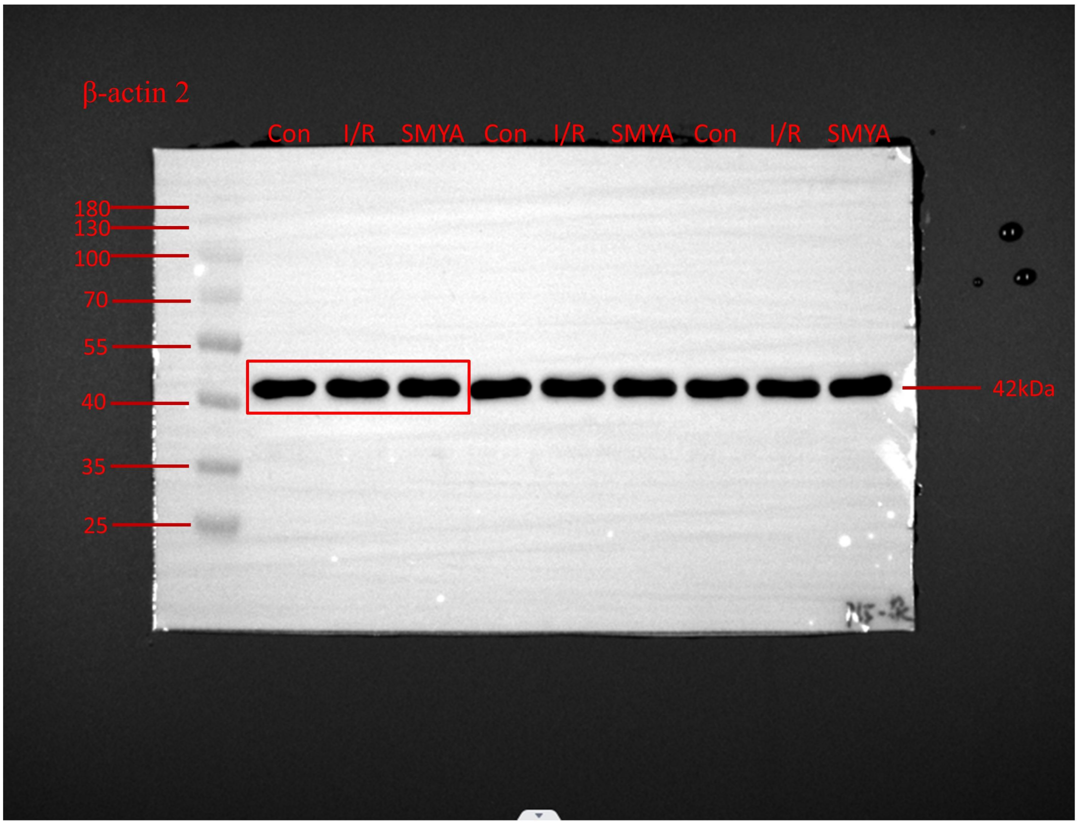

7. P65 Nucleus

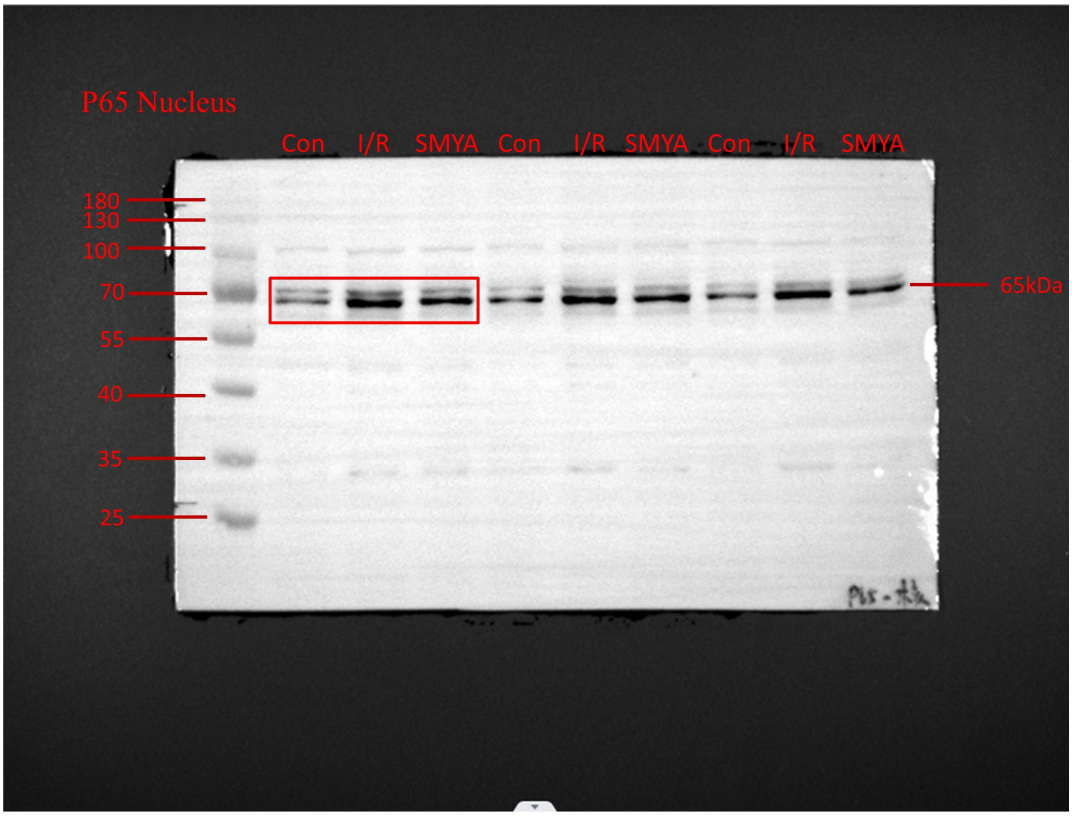

8. LaminB

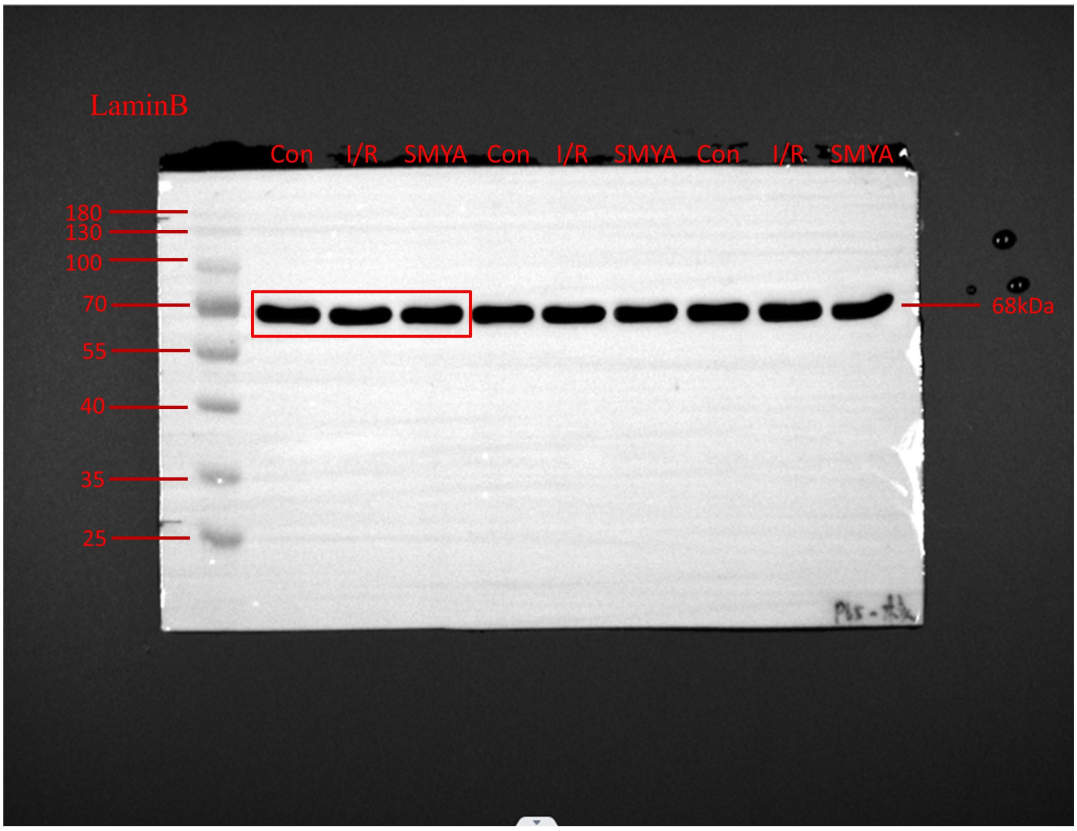

9. ZO-1

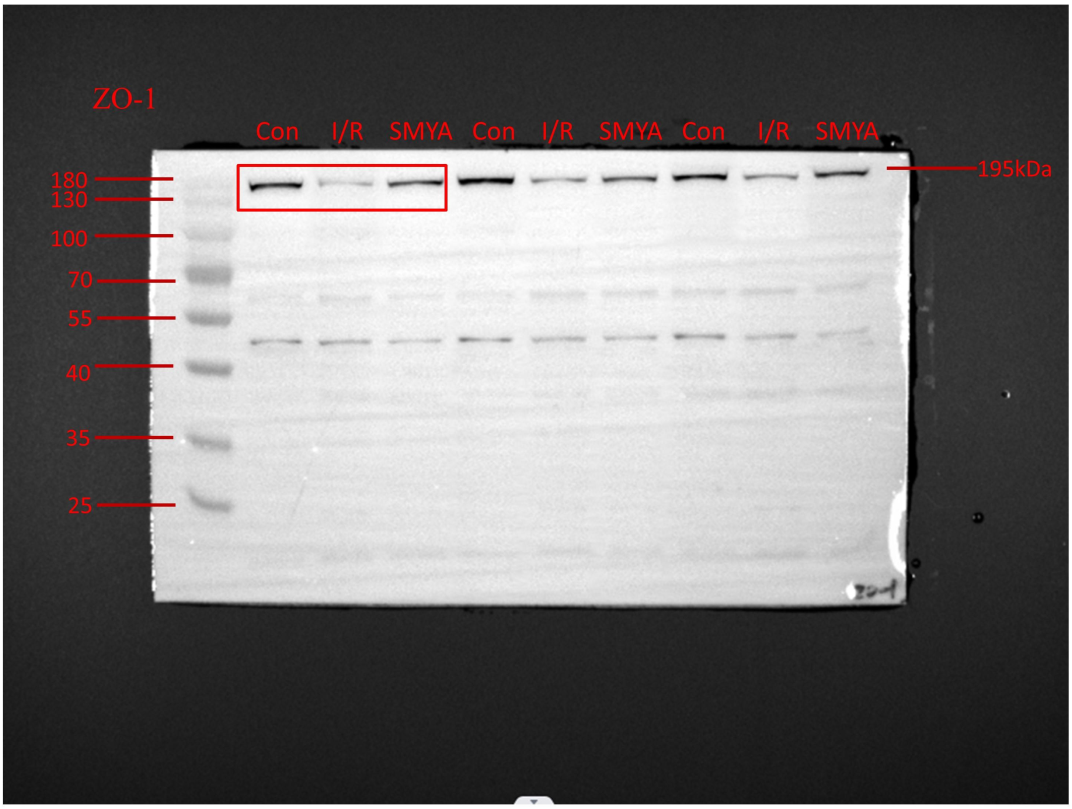

10. Occludin

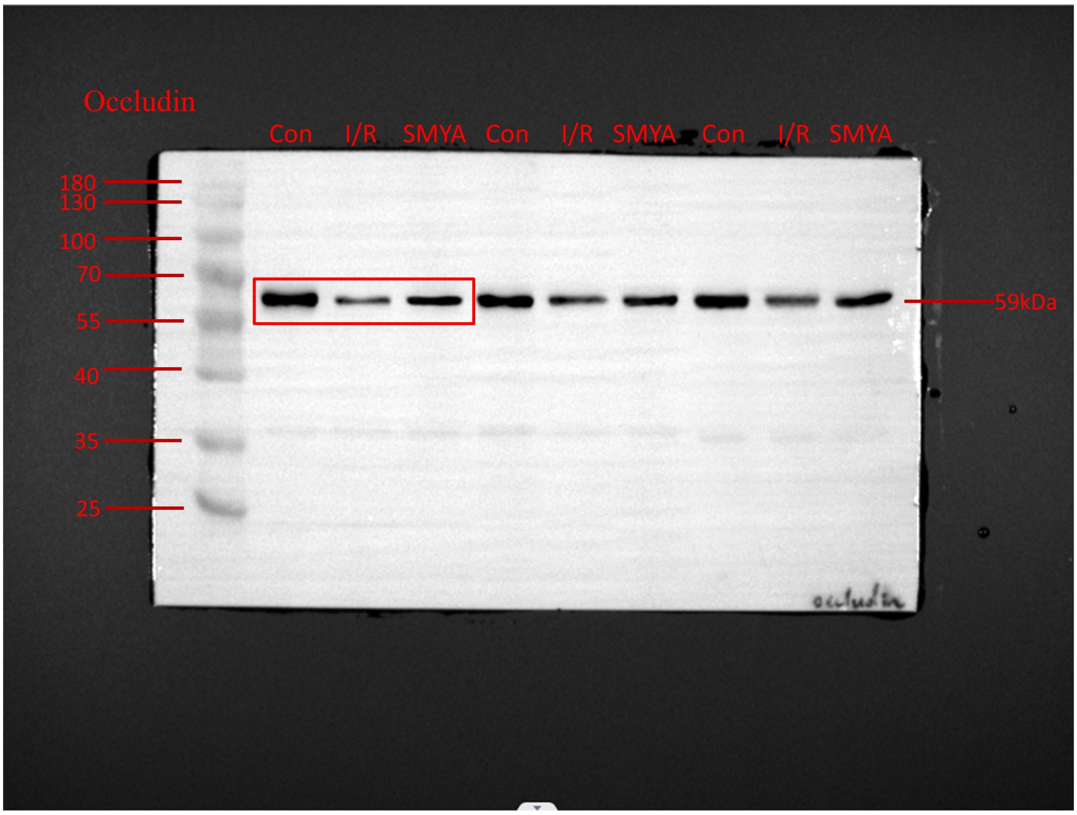

11. β-actin 3

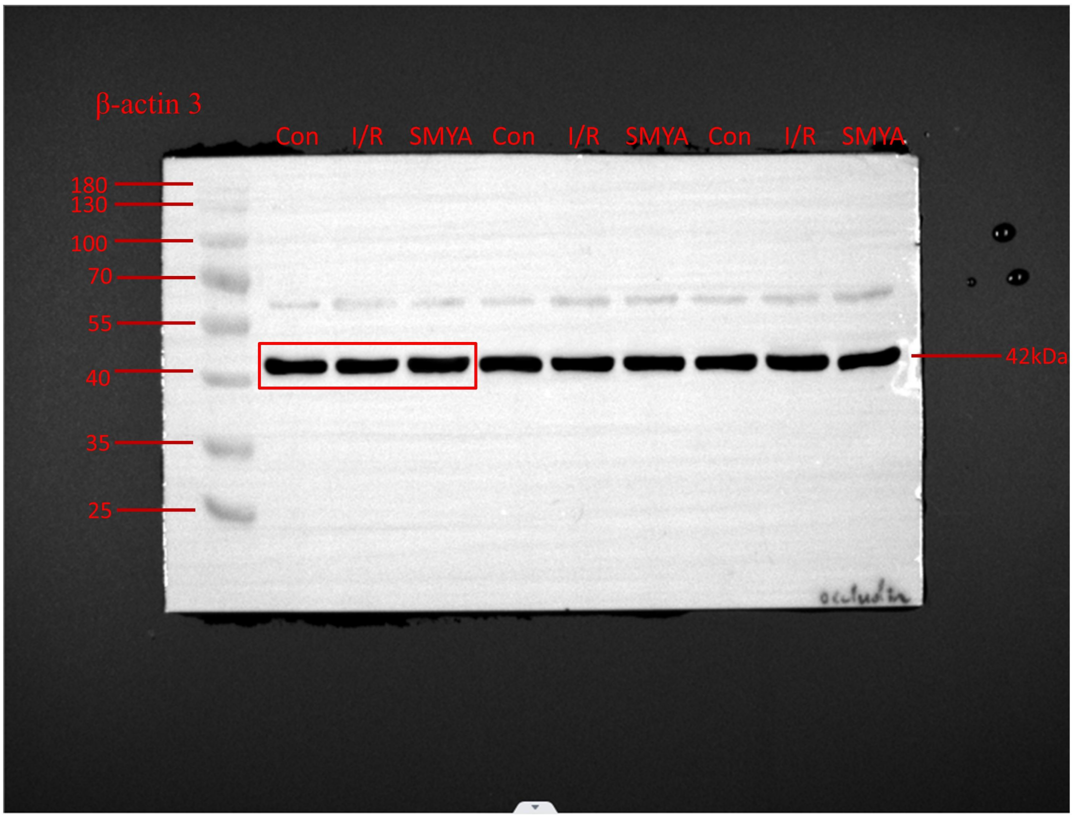

Supplement: Supplementary file 1 — Additional file 1. [file 12906_2023_4013_MOESM1_ESM.pdf]
